# Postpartum Depression Is Associated with Maternal Sociodemographic and Anthropometric Characteristics, Perinatal Outcomes, Breastfeeding Practices, and Mediterranean Diet Adherence

**DOI:** 10.3390/nu15173853

**Published:** 2023-09-04

**Authors:** Sousana K. Papadopoulou, Eleni Pavlidou, Antonios Dakanalis, Georgios Antasouras, Theofanis Vorvolakos, Maria Mentzelou, Aspasia Serdari, Aimilia-Lynn Pandi, Maria Spanoudaki, Olga Alexatou, Exakousti-Petroula Aggelakou, Constantinos Giaginis

**Affiliations:** 1 Department of Nutritional Sciences and Dietetics, School of Health Sciences, International Hellenic University, 57400 Thessaloniki, Greece; maryspan1@gmail.com; 2 Department of Food Science and Nutrition, School of the Environment, University of the Aegean, 81400 Myrina, Lemnos, Greece; elen.p.pavl@gmail.com (E.P.); g.antasouras@gmail.com (G.A.); maria.mentzelou@hotmail.com (M.M.); baltimoreargyrades@gmail.com (A.-L.P.); rd.olga.alexatou@gmail.com (O.A.); xeniaggelakou@hotmail.com (E.-P.A.); cgiaginis@aegean.gr (C.G.); 3 Department of Mental Health, Fondazione IRCCS San Gerardo dei Tintori, 20900 Monza, Italy; antonios.dakanalis@unimib.it; 4 School of Medicine and Surgery, University of Milano-Bicocca, 20900 Monza, Italy; 5 Department of Psychiatry, School of Health Sciences, University General Hospital of Alexandroupolis, Democritus University of Thrace, 68100 Alexandroupolis, Greece; tvorvola@med.duth.gr; 6 Department of Psychiatry and Child Psychiatry, School of Medicine, Democritus University of Thrace, 68100 Alexandroupolis, Greece; aserdari@yahoo.com

**Keywords:** postpartum depression, overweight, obesity, sociodemographic parameters, anthropometric parameters, perinatal outcomes, breastfeeding practices, type of delivery, Mediterranean diet, gestational diabetes, gestational weight gain

## Abstract

Postpartum depression, with a prevalence ranging between 14% and 25% worldwide, has been considered an urgent health concern that negatively affects both mothers’ and their infants’ health. Postpartum depression may negatively affect maternal sociodemographic and anthropometric parameters and lifestyle factors. Nutrition has recently been identified as a crucial factor for the management and co-treatment of postpartum depression. This survey aims to determine the possible association of postpartum depression with mothers’ socio-demographic and anthropometric characteristics, perinatal outcomes, breastfeeding practices, and Mediterranean diet (MD) adherence. Methods: This is a cross-sectional survey, which was performed on 3941 women during the postpartum period. Postpartum depression was assessed by the Edinburgh Postnatal Depression Scale (EPDS). Anthropometric parameters and perinatal outcomes were retrieved from mothers’ medical records. Sociodemographic data and breastfeeding practices were recorded by face-to-face interviews between enrolled mothers and trained personnel. Mediterranean diet adherence was assessed by MedDietScore. Both univariate and multivariate binary logistic regression were applied for analyzing our data. Results. Postpartum depression was significantly associated with lower educational level, Greek nationality, higher prevalence of multiparity and overweight/obesity postpartum, higher incidence of caesarean section and not breastfeeding, and lower levels of MD adherence. In multivariate analysis, postpartum depression was independently associated with mothers’ educational level, postpartum BMI status, type of delivery, breastfeeding practices, and MD adherence after adjusting for multiple confounding factors. Conclusions: This study has provided evidence that elevated MD compliance was related to a decreased risk of postpartum depression. Additionally, postpartum depression was associated with multiple sociodemographic and anthropometric parameters, perinatal outcomes, and breastfeeding practices. Future well-designed, prospective studies with high-quality methodology should be performed to obtain conclusive results.

## 1. Introduction

Postpartum depression constitutes an urgent health issue, which seems to adversely influence both mothers and their infant health. Postpartum depression manifests during the first four to six weeks after delivery or even until the first six months, a severe public health concern [1,2]. The prevalence of postpartum depression usually ranges from 14% to 25% [3,4,5]. Alarmingly enough, this prevalence has increased to 34% during the COVID-19 pandemic [6]. Untreated postpartum depression may negatively affect mothers’ mental and physical health. Worryingly, it also exerts a negative effect on children’s development and health [7]. It also negatively affects the relationship between mothers and children [8]. The most important risk factors of postpartum depression are low social support [9], poor family relationships [10], marital relationships [3], low financial status [11], and mental comorbidities [12,13]. Moreover, dealing with interpersonal difficulties also exert a major role [14,15].

The most usual symptoms of postpartum depression include depressive mood, inadequate sleep quality, energy loss, guilting moods, the tendency to become annoyed, anxiety, and suicidal feelings [16]. In addition, postpartum depression can highly disturb the connections and the interrelationships between the mothers and their newborns [16], leading to breastfeeding interruption even in the first days after delivery [17], resulting in child growth complications [18] and contributing to cognition and behavior dysfunctions in children of women suffered from postpartum depression [19,20]. Psychotherapy or antidepressant medication has been suggested by the American Psychiatric Association as a first-line treatment for mild-to-moderate postpartum depression [21,22,23]. The monoamine-based medication is broadly utilized as the standard therapy [24]. Selective serotonin reuptake inhibitors (SSRIs), serotonin and norepinephrine reuptake inhibitors (SNRIs), and tricyclic anti-depressants (TCAs) have been considered the most suitable and effective treatment approaches up to date [25,26].

Interpersonal psychotherapy constitutes a novel and effective therapeutic approach against postpartum depression, which can treat mental disorders through a physiological and psychosocial model [27,28]. This model has extensively been utilized in the management and monitoring of depression [29]. Recent studies have focused on diverse physiological aspects like peptide and steroid hormone modifications, which happen across gestation or the period after delivery [30,31]. Peptide and steroid hormone modifications may influence the hypothalamus-hypophysis–sex glands and the hypothalamus–hypophysis-adrenals glands axes in women. Abnormalities in these hormonal axes have been associated with mood-associated diseases across gestation and during the period after delivery [32].

There are currently several studies exploring the interrelationships between dietary habits and psychological and emotional behaviors, which have attracted the interest of researchers worldwide [33]. Several dietary patterns and specific foodstuffs have been investigated as complementary therapeutic agents in clinical practice to improve postpartum depressive symptoms in women who are at high risk. Results from recent studies have demonstrated that several nutrients are required for neurotransmitters’ biosynthesis, exerting a crucial biological effect on the nervous system. Therefore, they could positively influence several aspects of mental health [34]. In addition, nutrient deficiencies constitute a common phenomenon in the first months after delivery. This is mainly ascribed to the reasonably increased nutritional requirements of mothers’ bodies, across gestation and lactation periods [35]. Currently, the studies evaluating the relationship between nutrient consumption and/or serum concentrations of foodstuffs ingredients with antioxidant and/or anti-inflammatory properties throughout gestation with the prevalence of postpartum depression remain extremely limited.

In the last few years, several studies have documented that specific dietary patterns, foodstuffs and nutrients can exert antidepressant effects by diverse molecular mechanisms. These mechanisms exert strong effects against inflammation-related conditions and oxidative stress, enhancing the monoamine neurotransmitter biosynthesis, reducing the hyperactivity of the hypothalamus–hypophysis-adrenal axis, and modulating the microbiome-gut-brain axis [36,37,38,39]. Both nutrition and physical activity interventions seem to improve postpartum depressive symptoms. Moreover, combining diet and physical activity seems to reduce depressive symptoms in mothers who suffered from depression for a long time [40]. In this aspect, several evidence has documented that adopting healthy dietary habits by consuming vegetables and fruits, not following a pro-inflammatory diet such as junk and fast foods, and high meat product consumption could decrease the probability of depression symptomatology [41]. Certain surveys have also shown that several foodstuffs’ ingredients like folic acid, vitamins B6 and B12, polyunsaturated fatty acids, zinc, selenium, and magnesium may reduce the probability of developing depressive symptomatology [42]. 

A systematic review of older adults showed that the Mediterranean diet (MD) could reduce the risk of developing depression and improve depression symptomatology. This was ascribed to its beneficial effects against inflammation-related conditions and oxidative stress [43]. Another study has indicated that the MD pattern may decrease the probability of developing depression, ameliorating depressive symptomatology [44]. On the contrary, Western nutritional habits seem to enhance the likelihood of depression and increase the intensity of depressive symptoms in adolescents [44]. It has also been found that pro-inflammatory diets may raise the probability of developing depression. In contrast, anti-inflammatory and antioxidant diets like MD may reduce the risk of depression [45].

In this context, MD is a plant-based diet rich in anti-inflammatory and antioxidant components, which contains high amounts of vegetables, fruits, cereals, nuts and extra virgin olive oil, a modest intake of dairy products, seafood and poultry, and decreased intake of saturated fats and processed red meat products. The MD was initially invented by Ancel Keys in 1960 [46], and it has been recognized as one of the most explored and popular diets, attracting the interest of the scientific community worldwide [47]. MD can act as a preventive agent for various human disorders by lowering the risk of cardiovascular diseases, metabolic disorders, neurodegenerative diseases, cancer, and other diseases, including depression [48,49,50,51]. In a previous survey, we demonstrated that elevated MD compliance is highly related to greater cognition status and lower prevalence of depression symptoms in older adults [52]. A greater nourishment status has been related to a decreased likelihood of cognitive impairment and depression symptoms in older Greek adults [53].

Nevertheless, the currently available studies remain scarce concerning the investigation of the relation of maternal diet and lifestyle factors with the prevalence of postpartum depression. Moreover, even fewer studies have assessed the impact of MD on postpartum depression, while only a few studies have explored the association of postpartum depression with sociodemographic and anthropometric parameters and lifestyle factors. In this aspect, the current survey is one of the few studies that has aimed to assess the potential relation of postpartum depression with maternal socio-demographic and anthropometric parameters, perinatal outcomes, breastfeeding practices, and MD adherence and to evaluate for the first time whether higher levels of MD adherence could decrease the probability of developing postpartum depression.

## 2. Methods

### 2.1. Study Population

In the current survey, 6291 mothers were primarily assigned from 11 geographically diverse Greek areas, rural, urban and islands (Athens, Thessaloniki, Larissa, Kavala, Alexandroupolis, Ioannina, Patra, Kalamata, Crete, South and North Aegean). The inclusion criteria for the primary assignment were mothers with singleton childbirth before the enrollment, independently of parity. In multiparous mothers, merely the last pregnancy was considered. Assignment to the survey was from May 2016 to September 2020. Mothers’ recruitment was between the 3rd and the 6th month after delivery.

**Figure 1:**
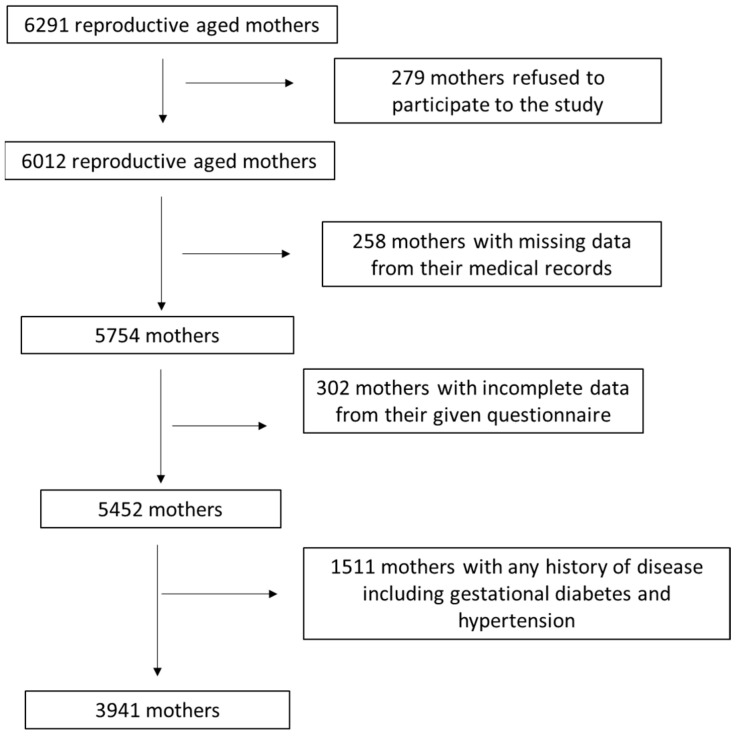
Flow chart of study enrolment.

A detailed description of study enrolment as a flow chart diagram is depicted in Figure 1. After applying relevant exclusion and inclusion criteria, 3941 reproductive-aged women were included in the final analysis, resulting in a final response rate equal to 62.6%.

The Ethics Committee of the University of the Aegean approved this study (ethics approval code: No. 12/14.5.2016, approval date: 14 May 2016) and was in accordance with the World Health Organization (52nd WMA General Assembly, Edinburgh, Scotland, 2000).

All enrolled mothers’ information was confidential, and all participants had no disease at the time of the study. They were informed about the survey’s aim and signed a consent form. Sample size calculation was performed using PS: Power and Sample Size calculator program. The randomization was conducted using a sequence of random binary numbers (i.e., 001110110, in which 0 represented enrolment and 1 not enrolment to the study). The computation of the power of our sample size showed a power of 88.5%.

### 2.2. Study Design

Relevant semi-quantitative questionnaires were used to collect the sociodemographic data, including age, educational level, economic status, nationality, marital status, employment status, smoking habits, and parity of the enrolled mothers by face-to-face interviews between the enrolled mothers and the trained personnel to minimize recall bias. The educational level was classified into three categories: (a) primary education, (b) secondary education, and (c) university studies. Financial status was categorized according to the yearly family income as: 0 < 5000 €; 1 5000–10,000 €; 2 10,000–15,000 €; 3 15,000–20,000 €; 4 20,000–25,000 €; 5 > 25,000 €. Economic status was further categorized as low for families with a yearly income of ≤10,000 €, medium for a yearly income of ˃10,000 € and ≤20,000 €, and high for an annual income of ˃20,000 €.

The measured body weight and height of the enrolled mothers were retrieved from their medical files to evaluate the Body Mass Index (BMI) before gestation. Body weight and height were also measured at the time of study to calculate postpartum BMI. Mothers’ weight was assessed using a Seca scale [Seca, Hanover, MD, USA], without shoes, to the near 100 g, while height was assessed using a portable stadiometer (GIMA Stadiometer 27335, Athens, Greece) with no shoes on, to the nearby 0.1cm. The WHO recommendations were applied to classify the assigned mothers as normal weight, overweight or obese [54,55].

Perinatal outcomes, including gestational weight gain (GWG), preterm childbirth, and type of delivery (vaginal or caesarean section), were retrieved from the mothers’ medical files. All women were tested for gestational diabetes mellitus using a standardized oral glucose tolerance test (OGTT) during gestation [56]. More to the point, a fasting OGTT next to 75 g glucose with a cut-off plasma glucose of ≥140 mg/dL after 2-h for the initial and following trimester at 24–28 weeks of gestation had been applied for all participating women [56]. Those women diagnosed with gestational diabetes, according to the above criteria, were excluded from the study. Based on Institute of Medicine (IOM) guidelines, the proposed gestational weight gain for underweight women before gestation (BMI < 18.5 kg/m^2^) ranges from 12.5 to 18.0 Kg, for normal-weight women (BMI: 18.5–24.9 kg/m^2^) from 11.6 to 16.0 Kg, for overweight mothers (BMI: 25.0–29.9 kg/m^2^) from 7.0–11.5 Kg and mothers affected by obesity (BMI ≥ 30.0 kg/m^2^) from 5–9 Kg [57]. The enrolled mothers were classified according to the above criteria into three categories: (a) those with lower than the recommended GWG, (b) those with normal GWG and (c) those with excess GWG.

Moreover, the assigned mothers responded if they adopted breastfeeding practices and whether they have applied exclusive breastfeeding for their infant for at least four months [58,59]. To minimize recall bias, the women responded if they had exclusively breastfed their infant for at least four months as at this exact period, they were guided to progressively include pulp foodstuffs in the eating practices of their paired infants, and therefore, they remembered more exactly this time point, which increases the reliability of their responses. On the contrary, women who adopted lactating attempts for lower periods could not respond with adequate reliability concerning the exact period of the lactating procedure [58,59].

The assigned mothers further stated whether they had a preterm childbirth (<37th week), and their responses were additionally cross-checked in comparison with their medical records to ensure more reliable data concerning the precise week of pregnancy that preterm childbirth was performed. Nevertheless, we noticed that some medical records did not contain the exact week of childbirth. Moreover, some of them were not in accordance with the enrolled women’s answers. Hence, preterm childbirth was classified as a binary variable, with childbirth categorized before or after the 37th week of pregnancy, which is considered moderate to late preterm [58,59].

Edinburgh Postnatal Depression Scale (EPDS) assessed postpartum mothers’ depressive symptomatology [60]. The EPDS includes ten short statements. A mother checks off one of four probable responses to how she has felt the previous week [60,61]. Most participants have simply completed the scale in about five minutes. Answers are scored 0, 1, 2 and 3 according to the intensity of symptoms. Items 3, 5 and 10 are inverse scored (i.e., 3, 2, 1, and 0). The overall score is obtained by combining collectively the scores for each of the ten items. Participants with a score >12 score probably suffered from depression, and it is recommended to demand medical support [60,61]. The scale shows whether the participants felt in the last week, and it is useful to complete the scale again after two weeks. In this aspect, we repeated the scale after two weeks to minimize potential bias. The enrolled woman checks off the answer, which is closer to how she has felt in the last seven days, with the complementary aid of the qualified personnel during the face-to-face interviews. Care should thoroughly be performed to prevent the probability of the mother discussing her responses with others [60,61].

Concerning MD assessment, the validated MedDietScore was used [62,63]. This questionnaire records the food frequency consumption of eleven selected foodstuffs groups based on the MedDietScore index. Every question has six probable responses, ranging between 0 and 5, which depend on the degree of compliance for every foodstuff group. The summation of the eleven responses results in a score between 0 and 55; the greater scoring represents increased MD compliance [62,63]. Concerning cereals, potatoes, fruits, vegetables, dairy and olive oil, the rates of six probable responses referred to daily consumption. Regarding legumes, fish, red meat and poultry, the rates of six probable answers referred to weekly consumption [62,63]. The 11th question evaluates wine drinking at a daily frequency with intermediate drinking (≤1 and ≤2 drinks/day for women and men, respectively; one drink = 100 mL = 12 g ethanol), being recognized as the greatest scoring [62,63].

The qualified personnel comprehensively described in detail to all enrolled women how they must complete the questionnaires through face-to-face interviews to minimize recall bias. The qualified personnel comprehensively described all the questions for each questionnaire to enhance the validity of the answers.

### 2.3. Statistical Analysis

Student’s *t*-test was used concerning the continuous variables, which followed the normal distribution. The Kolmogorov-Smirnov test was applied to evaluate whether each continuous variable followed a normal distribution. Chi-square was used for categorical variables. The quantitative variables that were normally distributed were expressed by mean value ± Standard Deviation (SD). Non-parametric analysis using the Mann-Whitney test was applied for non-normally distributed variables. The quantitative continuous variables not normally distributed were expressed by median value (Interquartile Range, IQR). The qualitative variables are reported as absolute or relative incidences. To evaluate whether postpartum depression may independently be related to sociodemographic and anthropometric parameters, perinatal outcomes and breastfeeding practices, multivariate binary logistic regression analysis was applied by adjustment for potential confounding factors. The Statistica 10.0 software, Europe, was used for the statistical analysis of the under-study data (Informer Technologies, Inc., Ham-burg, Germany).

## 3. Results

### 3.1. Descriptive Statistics of the Study Population

This cross-sectional study included 3941 mothers and was performed postpartum between 3 and 6 months after delivery. The mean age of the enrolled mothers was 33.2 ± 5.6 years old (range: 21–48 years old). 24.4% of the enrolled mothers have received primary education, 40.9% have completed secondary education, and 34.7% have graduated from a university. 46.3% of the assigned mothers exhibited low economic status, 45.0% had a medium economic status, and only 8.7% reported a high economic status. Most enrolled mothers had Greek nationality (95.7%), and only 4.3% had other nationality. Concerning their marital status, most of them (77.0%) were married, and 67.6% of them reported that they were employed at the time of study. 74.3% of the enrolled women were never smokers, and 25.7% were systematically smokers. 61.3% of the assigned study reported that this was their first delivery, and 38.7% stated that they had another one or two deliveries in the past.

According to pre-pregnancy BMI status, 74.5% were classified as normal weight, 18.8% were categorized as overweight, and 5.7% as obese. In the postpartum period, three to six months after delivery, 75.5% were classified as normal weight, 16.7% as overweight and 7.8% as obese. According to IOM criteria, 3.1% of the enrolled mothers had lower GWG than the recommended, 70.8% had normal GWG, and 26.1% exhibited excess GWG. Preterm childbirth was performed on 21.6% of the enrolled mothers. 55.8% of the assigned mothers gave a child by caesarean section, while 44.2% gave childbirth by vaginal delivery. Concerning breastfeeding practices for the assigned mothers, almost half of them (49.9%) exclusively breastfed their infant for at least four months, and 50.1% did not breastfeed their infant or breastfeed their infant for a considerably lower period. MD compliance was classified into very low, low, moderate, and high compliance, and each quartile included an almost similar number of enrolled mothers. Based on the EPDS classification, 12.1% of the enrolled mothers were diagnosed with postpartum depression.

### 3.2. Associations of Postpartum Depression with Sociodemographic Parameters of the Study Population

Postpartum depression was significantly more frequently developed in older than younger women (Table 1, *p* = 0.0012). Postpartum depression was significantly associated with lower levels of education and worse family economic levels (Table 1, *p* ˂ 0.0001 and *p* = 0.0001, respectively). Greek mothers exhibited a considerably greater incidence of postpartum depression than mothers of other nationalities (Table 1, *p* = 0.0117). Women with more than one delivery had a significantly greater incidence of postpartum depression than those in which the present delivery was the first (Table 1, *p* = 0.0307). Marital and employment status and smoking habits were unrelated to the prevalence of postpartum depression (Table 1, *p* > 0.05).

### 3.3. Associations of Postpartum Depression with Anthropometric Parameters and Perinatal Outcomes of the Study Population

Postpartum depression was significantly related to elevated incidence of mothers overweight and obese at the time of study 3–6 months postpartum (Table 1, *p* < 0.0001). 32.3% of mothers with postpartum depression exhibited overweight or obesity, whereas this percentage decreased to 23.4% in mothers without postpartum depression (Table 1). In contrast, pre-pregnancy BMI status did not correlate with postpartum depression (Table 1, *p* > 0.05). Postpartum depression was also significantly related to a higher incidence of mothers’ excess GWG above the recommended IOM criteria (Table 1, *p* = 0.0051). Postpartum depression did not show any relation with preterm childbirth (Table 1, *p* > 0.05).

Postpartum depression was considerably more frequently developed in mothers giving birth by caesarean section than those giving birth by vaginal delivery (Table 1, *p* = 0.0001). Postpartum depression was significantly related to a lower prevalence of exclusive breastfeeding for at least four months (Table 1, *p* < 0.0001). Lastly, postpartum depression was significantly related to a lower incidence of adopting MD at higher levels (Table 1, *p* < 0.0001).

### 3.4. Multivariate Analysis for Postpartum Depression by Adjusting for Several Confounders

In multivariate binary logistic analysis, postpartum depression was significantly independently related to mothers’ educational level, postpartum BMI status, GWG, type of delivery, breastfeeding practices and MD compliance (Table 2, *p* < 0.05). In contrast, postpartum depression was not independently related to mothers’ age, economical status, nationality, marital and employment status, smoking habits, parity, pre-pregnancy BMI status, and preterm childbirth (Table 2, *p* > 0.05).

Women with postpartum depression exhibited a 38% higher probability of having lower education (Table 2, *p* = 0.0244). Postpartum depression was significantly related to a 95% greater probability of developing overweight and obesity during the postpartum period (Table 2, *p* = 0.0198). Postpartum depression was significantly associated with a 44% higher likelihood of developing excess GWG (Table 2, *p* = 0.0107). Moreover, postpartum depression showed a 93% higher probability of giving childbirth by caesarean section (Table 2, *p* = 0.0087). Women with postpartum depression exhibited a more than 2-fold lower likelihood of exclusively breastfeeding for at least four months (Table 2, *p* = 0.0032). Lastly, postpartum depression was significantly associated with a more than 2-fold lower probability of adopting MD at higher levels (Table 2, *p* = 0.0005).

## 4. Discussion

Postpartum depression has been recognized as the most common disease in the first months after delivery. In contrast, untreated postpartum depression has been shown to exert negative long-term effects for both mother and child. Substantial evidence has demonstrated that nutritional habits can exert a crucial impact on depression development and progression, as well as on the intensity of depression symptomatology in the general population [64,65]. Concerning postpartum depression, there is merely a small number of studies. These studies have specifically explored the relationship between mothers’ dietary habits during the postpartum period and the development of depression in the first week and months after delivery. The first months after delivery constitute a period of elevated nutritional needs and nutrient deficiencies, associated with higher demands for breastfeeding and replacement of depleted stores from gestation and delivery, which could elevate the risk of depression [66]. In addition, physical and mental changes, including emotional, behavioral, and cognitive re-adjustments related to novel roles and responsibilities, can affect the mothers during the first months after delivery, which could exert a negative impact on both dietary habits and mood behavior [67].

Moreover, mothers with inadequate nutrition and absence of prenatal medical care, as well as smokers and alcohol-use mothers, have been related to a higher risk of developing psychological distress [68,69,70]. Many studies have shown that higher compliance to healthier nutritional habits in the first months after delivery was related to a reduced probability of postpartum depression [71]. More to the point, the Traditional-Indian-Confinement” diet, which includes ethnic bread, legumes and pulses, whole milk, Indian herbs and seed herbs, and butter/ghee, was found to a suitable dietary pattern that has been related to less depressive symptomatology at three months after delivery [72]. Higher compliance to the “Soup-Vegetables-Fruits” diet, similar to a healthy nutritional pattern, has also shown a correlation trend with a lower prevalence of developing depressive symptomatology [72]. Interestingly, Chatzi et al. identified two distinctive nutritional patterns, termed ‘healthy’ and ‘Western’, in pregnant women in Greece. Notably, they showed that the ‘healthy’ pattern exerted a preventing effect against depression symptomatology at 8–10 weeks after delivery [73]. Accordingly, Vilela et al. conducted a cohort study in Brazil, showing that a healthy nutritional pattern may protect against antenatal depression [74].

On the other hand, Pina-Camacho et al. did not find any relationship between unhealthy nutritional habits, assessed by principal component analysis (PCA), and postpartum depression [75]. Leung et al. assessed the mean, range, and percent of Recommended Daily Allowance in consuming several B vitamins, Vitamin D, omega-3 PUFAs, iodine, iron, zinc, and selenium [76]. Amongst the above nutrients, merely selenium consumption was shown to have a positive impact against depression at 12 weeks after delivery [76].

Several studies applying PUFAs supplementation did not find evidence of an association with postpartum depression [77,78,79,80]. However, other clinical studies have revealed that healthy nutritional patterns, which include a combination of multivitamin consumption, seafoods and PUFAs consumption, calcium, zinc, and probable selenium, may exert various beneficial effects [77,78,79,80]. Judge et al. performed a study including 42 individuals who have received either docosahexaenoic acid (DHA) in fish oil or a placebo within the period of gestation and have shown that DHA consumption reduced the prevalence of postpartum depression in the intervention group [81]. Miyake et al. in Japan have also provided evidence for possible beneficial health effects of overall seafoods intake as well as DHA and eicosapentaenoic acid (EPA) consumption. On the other hand, elevated consumption of total fat and saturated fat were harmful risk factors for developing depression symptomatology [82]. Another recent study has shown that higher consumption of fruits, reduced consumption of red meat and subproducts and higher MD compliance were related to a reduced probability of developing depressive symptoms in the first weeks or months after delivery [83]. Higher levels of MD compliance during gestation have also been related to a lower prevalence of depression symptomatology and a reduced likelihood of developing postpartum depression [84,85,86,87,88,89]. There is not enough data to support that nutritional habits can affect the probability of developing depressive symptoms, while several controversies between the existing studies have appeared. Moreover, the existing results remain inconsistent and inconclusive.

Based on the above currently existing data, there is merely a small number of studies exploring the potential effects of a healthy nutritional pattern on the probability of developing depressive symptoms, and even fewer studies assessed the potential effects of MD adherence or its foodstuffs’ components in the risk of postpartum depression. In this aspect, the current survey constitutes the first cross-sectional study assessing the impact of MD compliance on the likelihood of developing depressive symptoms. Additionally, we have examined diverse maternal sociodemographic and anthropometric characteristics, perinatal outcomes, and breastfeeding practices in association with the risk of developing depressive symptoms in the first months after delivery. Our study has demonstrated that a higher incidence of postpartum depression was significantly associated with lower educational level, worse economic status, overweight/obesity postpartum, excessive GWG, higher risk of caesarean section delivery, not exclusive breastfeeding, and lower levels of MD adherence. The above results are in accordance with several previous findings [15,90,91]. In line with our results, a meta-analysis involving 100,438 participants has demonstrated that higher or lower than the IOM recommended GWG was considerably related to an increased probability of postpartum depression, highlighting the requirement of reinforcing the prevention of abnormal GWG during gestation to enhance and protect mothers’ and infants’ health [92].

In accordance with previous studies, it should be emphasized that greater levels of MD compliance have been related to a more than 2-fold lower risk of postpartum depression, providing evidence that this dietary pattern may effectively prevent the development of postpartum depression or even reduce its symptoms. Accordingly, exclusive breastfeeding has been associated with more than 2-fold reduced probability of postpartum depression, which is in line with the beneficial effects of breastfeeding in both the mother and their infants [58,93]. Our results concerning maternal sociodemographic and anthropometric characteristics and perinatal outcomes also agree with several previous substantial studies [94,95,96]. Notably, a well-designed meta-analysis has documented that mothers who have exclusively breastfed their infants have exhibited a 53% decreased likelihood of postpartum depression than those who have never breastfed their infants. In addition, mothers who have exclusively breastfed their infant showed an 8% decreased probability of developing depressive symptoms after delivery than those who have only partially breastfed their infant [93].

Furthermore, an increased prevalence (27.5%) of preterm birth was recorded among the enrolled mothers, while the mean age of the enrolled mothers increased (33.2 ± 5.6 years old). According to the Eurostat data, the mean age of women who had a first child has steadily increased to 29.4 years in 2019 [97]. Notably, the mean age of women with a first child in our country is among the highest (30.6 years old) in the European Union countries [97]. This may be ascribed to the fact that 37.8% of the enrolled mothers in our study have given birth to their second or third child. Indeed, when we separated nulliparous from multiparous mothers, the mean age of mothers giving birth to their first child was 31.3 ± 5.5 years, which is close to the Eurostat data [97]. Notably, in 2022, the mean age of mothers in Luxembourg reached 32.2 years, the highest amongst European countries, whereas Bulgaria was identified as the country with the youngest mean age at 27.8 years [98]. Concerning preterm birth, in 2010, about 14.9 million newborns were delivered preterm, corresponding to 11.1% of all childbirths globally, varying from about 5% in some European countries to 18% in certain countries of Africa [99]. It has been supported that classifying preterm childbirths that are spontaneous from those that are provider-initiated is of great importance in controlling the increasing trends related to the high prevalence of caesarean sections [99]. Thus, the increased prevalence of preterm childbirth in our enrolled mothers may be ascribed, at least partially, to the increased incidence of caesarean sections in our study.

Another concern in our study is the increased prevalence (55.8%) of caesarean sections. This may be attributed, at least in part, to the fact that many cases of caesarean section in our study population may be elective. It should be noted that the most common cause of the primary behind women’s request to deliver by caesarean delivery is the pathological fear related to the labor procedure, recognized under the scientific term “tocophobia” [100,101]. In this context, it has been demonstrated that the prevalence of cesarean sections has gradually been elevated during the last three decades, and it has currently ranged from 0.4% to 65% [100,101]. Notably, a considerable prevalence of pregnant women, varying from 1% to 20%, demand a caesarean section without any medical reason [100,101].

The present survey has several advantages as it was conducted on an adequately representative sample of mothers enrolled in diverse areas of Greece. The study population was quite large and included only Caucasian women living in 11 diverse Greek regions; therefore, its representativeness may be recognized as quite adequate. Hence, our findings could be generalized to Caucasian European populations of other nationalities. Moreover, our study is one of the few studies that has investigated the relation of MD compliance during the postpartum period in association with multiple sociodemographic and anthropometrical characteristics, perinatal outcomes, and breastfeeding practices. Another advantage of our study is that face-to-face interviews between the participating mothers and the qualified personnel were performed to reduce recall bias. The detailed guidelines and the thorough demonstration of the questions provided by the face-to-face interviews may also reduce potential recall bias, increasing the accuracy and reliability of responses.

Moreover, our study population contained only healthy mothers with no history of any severe disease, including gestational diabetes and pregnancy-induced hypertension, to avoid comorbidities confounding effects. We also examined whether postpartum depression exerts an independent effect by adjustment for many potential confounding factors. Finally, we used the Edinburg Postpartum Depression Scale (EPDS), well-recognized worldwide as the gold standard for screening to detect postpartum depression [102].

The understanding of the current results should also take into consideration some disadvantages in mind. The cross-sectional design of the study reduces the likelihood of supporting conclusive results and suffers from the possible risk of recall biases, particularly concerning the self-reported questions, even if we have performed face-to-face interviews. Thus, no definitive conclusions about causality could be derived because of our study design. However, self-reported data have extensively been applied in epidemiological studies, showing great consistency and validity in predicting several outcomes. A second disadvantage of our survey is that BMI was applied to classify the enrolled mothers as overweight or obese before gestation and postpartum. In this aspect, body fat mass and distribution should directly be determined and included in future studies to extend and validate our results. In addition, we recognize the possibility of unmeasured confounding factors such as mental health status, sleep disturbances, eating disorders, and the physical activity of the enrolled mothers despite our thorough approach to confounding adjustment. Hence, although we have performed adjustments for several confounders, it remains probable that residual confounding may influence our results. In this aspect, a substantial clinical study has explored the regulatory roles of sleep and depression on the relationships amongst stress, fat consumption, and fruit and vegetable consumption concerning low-income, overweight, and obese pregnant women by trimesters. This study has supported evidence that stress may be related to depression during pregnancy [103].

Moreover, depending on the pregnancy trimester, night-time sleep disruptions, sleep quality, and sleep latency may be associated with depression [103]. Furthermore, insulin resistance has been identified as a communal pathologic mechanism between depression and type 2 diabetes, and it could be considered a potential confounding factor in study populations, including women with type 2 diabetes [104]. Finally, a last disadvantage of our study is that the mothers’ recruitment was not performed at the one-time point but in a period between 3rd and 6th month after delivery, which may affect certain variables such as dietary habits expressed by MD adherence or postpartum BMI, which also might be affected by the exact time span of the study.

## 5. Conclusions

The current survey has supported evidence that a higher prevalence of postpartum depression was independently associated with lower educational level, postpartum overweight/obesity, excess GWG, higher risk of caesarean section delivery, and not exclusive breastfeeding. Future studies on a larger sample size, including adipose tissue body distribution (e.g., abdominal obesity), will consider potential comorbidities such as mental disturbances. and eating disorders should be performed. Well-designed, prospective, and population-based studies are strongly recommended to obtain conclusive results.

## Figures and Tables

**Table 1:** Associations of postpartum depression with mothers’ socio-demographic and anthropometric characteristics, perinatal outcomes, breastfeeding practices, and Mediterranean Diet adherence.

Characteristics (*n* = 3941)	Postpartum Depression (*n*, %)		
	No 3464 (87.9%)	Yes 477 (12.1%)	*p*-Value
**Age (mean ± SD; years)**	32.7 ± 5.4	33.9 ± 53	*p* = 0.0012
**Education level (*n*, %)**			*p* ˂ 0.0001
Primary education	643 (18.6%)	318 (66.7%)	
Secondary education	1484 (42.8%)	127 (26.6%)	
University studies	1337 (38.6%)	32 (6.7%)	
**Family economic status (*n*, %)**			
Low	1625 (46.9%)	201 (42.1%)	*p* = 0.0001
Medium	1518 (43.8%)	256 (53.7%)	
High	321 (9.3%)	20 (4.2%)	
**Nationality (*n*, %)**			*p* = 0.0117
Greek	3305 (95.4%)	467 (97.9%)	
Other	159 (4.6%)	10 (2.1%)	
**Marital status (*n*, %)**			*p* = 0.5698
Married	2661 (76.8%)	372 (78.0%)	
Other	803 (23.2%)	105 (22.0%)	
**Employment (*n*, %)**			*p* = 0.2276
Employed	2330 (67.3%)	334 (70.0%)	
Unemployed	1134 (32.7%)	143 (30.0%)	
**Smoking habits (*n*, %)**			*p* = 0.4010
No smokers	2582 (74.5%)	347 (72.7%)	
Smokers	882 (25.5%)	130 (27.3%)	
**Parity (*n*, %)**			*p* = 0.0307
Nulliparity	2146 (62.0%)	271 (56.8%)	
Multiparity	1318 (38.0%)	206 (43.2%)	
**Pre-pregnancy BMI status (*n*, %)**			*p* = 0.8239
Normal weight	2618 (75.6%)	356 (74.6%)	
Overweight	651 (18.8%)	91 (19.1%)	
Obese	195 (5.6%)	30 (6.3%)	
**Postpartum BMI status (*n*, %)**			*p* ˂ 0.0001
Normal weight	2656 (76.7%)	323 (67.7%)	
Overweight	539 (15.6%)	117 (24.5%)	
Obese	269 (7.8%)	37 (7.8%)	
**Gestational weight gain (*n*, %)**			*p* = 0.0051
Low	114 (3.3%)	10 (2.1%)	
Normal	2474 (71.4%)	315 (66.0%)	
Excess	876 (25.3%)	152 (31.9%)	
**Preterm birth (<37th week, *n*, %)**			*p* = 0.9999
No	2716 (78.4%)	374 (78.4%)	
Yes	748 (21.6%)	103 (21.6%)	
**Type of delivery (*n*, %)**			*p* = 0.0001
Vaginal	1569 (45.3%)	172 (36.1%)	
Caesarean section	1895 (54.7%)	305 (63.9%)	
**Exclusive breastfeeding (*n*, %)**			*p* ˂ 0.0001
No	1674 (48.3%)	302 (63.3%)	
Yes	1790 (51.7%)	175 (36.7%)	
**Mediterranean Diet adherence (*n*, %)**			*p* ˂ 0.0001
Very low	807 (23.3%)	174 (36.5%)	
Low	854 (24.6%)	126 (26.4%)	
Moderate	903 (26.1%)	103 (21.6%)	
High	900 (26.0%)	74 (15.5%)	

**Table 2:** Multivariate analysis for postpartum depression by adjustment for several confounding factors.

Characteristics	Postpartum Depression (No vs. Yes)	
	RR * (95% CI **)	*p*-Value
**Age** (Below vs. Over mean value)	1.32 (0.62–2.1)	*p* = 0.0819
**Education level** (Primary education vs. Secondary education and university studies)	1.38 (1.02–1.73)	*p* = 0.0244
**Family economic status** (High vs. Low or medium)	1.19 (0.59–1.88)	*p* = 0.0936
**Nationality** (Greek vs. Other)	1.06 (0.39–1.88)	*p* = 0.3134
**Marital status** (Married vs. Other)	0.93 (0.21–1.74)	*p* = 0.7501
**Employment** (Employed vs. Unemployed)	1.05 (0.27–1.83)	*p* = 0.5812
**Smoking habits** (No vs. Yes)	1.16 (0.45–1.98)	*p* = 0.7129
**Parity** (Nulliparity vs. Multiparity)	1.38 (0.67–2.12)	*p* = 0.2733
**Pre-pregnancy BMI status** (Normal weight vs. Overweight & obese)	1.11 (0.27–2.01)	*p* = 0.8159
**Postpartum BMI status** (Normal weight vs. Overweight & obese)	1.95 (1.58–2.23)	*p* = 0.0198
**Gestational weight gain** (Low and normal vs. Excess)	1.44 (1.11–1.79)	*p* = 0.0107
**Preterm birth** (No vs. Yes)	1.06 (0.45–1.89)	*p* = 0.7897
**Type of delivery** (Vaginal/Caesarean section)	1.93 (1.62–2.1.27)	*p* = 0.0087
**Exclusive breastfeeding** (Yes/No)	2.44 (2.25–2.514)	*p* = 0.0031
**Mediterranean Diet adherence** (Moderate + High/Very low + Low)	2.51 (2.33–2.68)	*p* = 0.0005

* Relative Risk: RR. ** CI: Confidence Interval.

## Data Availability

Available upon request to the corresponding author.
